# An fMRI study of concreteness effects in spoken word recognition

**DOI:** 10.1186/1744-9081-10-34

**Published:** 2014-09-30

**Authors:** Tracy Roxbury, Katie McMahon, David A Copland

**Affiliations:** Royal Brisbane and Women’s Hospital, University of Queensland Centre for Clinical Research, Level 3 Building 71/918, Herston, 4029 QLD Australia; School of Health and Rehabilitation Sciences, University of Queensland, St Lucia, 4072 QLD Australia; Centre for Advanced Imaging, University of Queensland, St Lucia, 4072 QLD Australia; Clinical Centre for Research Excellence in Aphasia Rehabilitation, Brisbane, Australia

**Keywords:** Language, fMRI, Neuroimaging, Auditory lexical decision, Concrete, Abstract

## Abstract

**Background:**

Evidence for the brain mechanisms recruited when processing concrete versus abstract concepts has been largely derived from studies employing visual stimuli. The tasks and baseline contrasts used have also involved varying degrees of lexical processing. This study investigated the neural basis of the concreteness effect during spoken word recognition and employed a lexical decision task with a novel pseudoword condition.

**Methods:**

The participants were seventeen healthy young adults (9 females). The stimuli consisted of (a) concrete, high imageability nouns, (b) abstract, low imageability nouns and (c) opaque legal pseudowords presented in a pseudorandomised, event-related design. Activation for the concrete, abstract and pseudoword conditions was analysed using anatomical regions of interest derived from previous findings of concrete and abstract word processing.

**Results:**

Behaviourally, lexical decision reaction times for the concrete condition were significantly faster than both abstract and pseudoword conditions and the abstract condition was significantly faster than the pseudoword condition (p < 0.05). The region of interest analysis showed significantly greater activity for concrete versus abstract conditions in the left dorsolateral prefrontal cortex, posterior cingulate and bilaterally in the angular gyrus. There were no significant differences between abstract and concrete conditions in the left superior temporal gyrus or inferior frontal gyrus.

**Conclusions:**

These findings confirm the involvement of the bilateral angular gyrus, left posterior cingulate and left dorsolateral prefrontal cortex in retrieving concrete versus abstract concepts during spoken word recognition. Significant activity was also elicited by concrete words relative to pseudowords in the left fusiform and left anterior middle temporal gyrus. These findings confirm the involvement of a widely distributed network of brain regions that are activated in response to the spoken recognition of concrete but not abstract words. Our findings are consistent with the proposal that distinct brain regions are engaged as convergence zones and enable the binding of supramodal input.

## Background

How conceptual knowledge is represented and organised in the brain is a question that remains a point of contention. Previous language studies have attempted to unravel some of the complexities surrounding the organisation and access of semantic conceptual representations and have investigated processing differences between concrete and abstract words. Concrete words such as ‘hospital’ are grounded in sensory-motor experiential knowledge while abstract words such as ‘knowledge’ refer more to verbally encoded concepts [1]. Imageability is often associated with concreteness and these constructs have been treated synonymously [2]. However, we acknowledge that they are distinct psycholinguistic concepts [3, 4] and for the purposes of this study, focus on processing differences associated with concrete versus abstract words in spoken word recognition.

Behavioural evidence from healthy individuals has demonstrated that concrete items are processed faster and more accurately than abstract items [1, 5–7]. This processing advantage or concreteness effect has been reported in people with aphasia [8], deep dyslexia [9–11] and semantic dementia [12]. However, the reverse effect, where abstract words are able to be processed more efficiently than concrete words has also been observed [4, 13–16]. This reversal of behavioural effects has been used to suggest the possible independent storage of these conceptual representations.

Theories of semantic memory have been developed to explain concreteness effects. Two prominent and competing accounts are the dual coding theory [5] and the context availability theory [17]. Both theories predict a superior processing advantage for concrete words due to the increased efficiency of processing systems associated with concrete words. Dual coding theory proposes that two structurally distinct coding systems, a verbal code and a nonverbal, perceptual code, are selectively engaged in response to different types of stimuli. According to this theory, both abstract and concrete words are processed in the verbal system but concrete words are able to recruit additional representations in a nonverbal, imagery-based system. As a result of this dual representation, concrete words are able to be processed more efficiently, resulting in the processing advantage.

In comparison, the context availability theory [17] suggests that the efficiency with which a word is able to be processed is due to the amount of available context associated with the target. Available context can be thought of in terms of the additional discourse/contextual information preceding the target word [18] or the specific semantic knowledge about the target word which is individual to each person [19]. Words with richer available context, such as concrete concepts, will be processed more efficiently since they have access to a larger network of contextual semantic associations thus resulting in the observed concreteness effect. Abstract words do not have the same level of context surrounding them as their meaning is largely derived from other verbal codes and as such, they are not able to be processed as efficiently.

Both theories acknowledge that abstract words are associated with a reduced set of semantic associations compared to concrete words either in terms of imagery or available context [20]. However, differences between the models are centred around the underlying neural substrates which contribute to concreteness effects. Dual coding theory proposes qualitatively distinct systems which are differentially activated such that abstract words and concrete words are processed in the same verbal system in the language dominant left hemisphere but concrete words are able to utilize the additional nonverbal, imagery-based system in the right hemisphere. As a result, concrete words should have a more bilateral representation [1, 6]. Context availability theory on the other hand proposes quantitative differences within a single system located in the verbal left hemisphere. Both abstract and concrete words will utilize the same system but activation for concrete items will be more extensive due to their richer context availability.

More recent theories of semantic memory have combined neuroimaging and behavioural data to explain conceptual processing. One such account by Binder and Desai [21] describes a modified embodiment theory (‘embodied abstraction’) which proposes that conceptual information is processed by modality-specific and heteromodal convergence zones. The convergence zones are located in temporal and inferior parietal regions situated between sensory, motor and affective systems. Modality-specific representations initially develop in response to repeated perceptual experiences and these modal representations converge with high-level heteromodal convergence zones which serve to bind the representations from different modalities [21]. Concept representations are differentially processed based on how familiar they are and the amount of perceptual and contextual information that is available about the target [21]. Thus, as concrete words have strong sensory-motor representations, they would be expected to elicit increased activation compared to abstract words due to their richer set of conceptual features.

It is clear that embodiment theories can account for the representation of concrete concepts but the case for abstract concepts is less obvious. Recent studies have investigated the semantics associated with abstract words [4, 22, 23]. Vigliocco et al. [23] and Kousta et al. [4] have proposed an extended theory of embodiment and applied a framework which includes abstract words and meaning. They suggest that semantic conceptual representations of concrete and abstract words are acquired through experiential and linguistic knowledge but to different degrees. They argue that concrete concepts are strongly associated with experiential knowledge based in sensory-motor experiences while abstract words and meanings are strongly grounded in linguistic *as well as* experiential knowledge [4, 23]. However, it is the type of experiential knowledge associated with the two word types that differs. While concrete words are embodied through sensorimotor experiential knowledge, abstract words are also embodied but their embodiment occurs instead through the underlying affective and emotional experiential knowledge associated with abstract words [4, 23, 24].

Increasingly, neurophysiological evidence has been used to buttress accounts of concreteness and define putative brain regions associated with concrete and abstract conceptual processing. Both auditory and visual modality paradigms have been employed but visual modality tasks have been the most utilized and have included visual recognition [25, 26], visual semantic similarity decisions [27–29], visual semantic categorisation tasks [20, 30] and visual lexical decision tasks [6, 24, 31–34]. Auditory modality studies meanwhile have utilized passive listening [35, 36] and mental imagery generation [37, 38] but it is unclear how these latter studies relate to spoken word recognition versus the retrieval of concrete versus abstract representations. In general, findings from both visual and auditory studies investigating concrete and abstract words have been highly variable and far from conclusive. Some studies have reported greater left hemisphere activity associated with concrete word processing [31, 36, 37, 39] while others have shown more of a bilateral pattern of activation for concrete words [6, 27] and greater left hemisphere activation for abstract words [6, 27] which has been used to support theories of dual coding. Meanwhile, other studies have not shown activation for concrete words in either hemisphere but instead elicited activity for abstract words only and this varied from the left [28] to right [32] to bilateral hemispheres [20, 30, 33, 40]. These findings do not support either context availability theory or dual coding theory as neither suggests greater activity for abstract terms should occur. The discrepant findings on concreteness effects have been largely attributed to differences in methodology, modality of input [41], baseline contrasts, and differences in imaging techniques.

Recent meta-analyses by Binder et al. [42] and Wang et al. [43] have combined findings from previous studies on concreteness effects with the aim of clarifying some of the inconsistent results. The meta-analysis by Binder et al. [42] included 17 studies on concrete and abstract processing and identified 113 overlapping foci associated with perceptual (concrete) processing and 34 for verbal (abstract) processing. Regions associated with concrete processing were located in bilateral angular gyrus (AG), left posterior cingulate, left dorsomedial prefrontal cortex and left mid fusiform while left inferior frontal gyrus (IFG), largely pars orbitalis and anterior superior temporal sulcus (aSTS) were associated with abstract processing. The meta-analysis on concrete word processing by Wang et al. [43] was based on 19 studies, ten of which were also included in the Binder et al. meta-analysis [42]. Concrete words showed more activation in left precuneus, left posterior cingulate, left fusiform and left parahippocampal regions while abstract processing was associated with left IFG, left middle temporal gyrus (MTG) and left superior temporal gyrus (STG).

One type of task commonly used to explore putative cognitive processing mechanisms associated with word recognition and semantic processing is a lexical decision task which is the focus of the present study. Lexical decisions have been shown to produce reliable semantic effects when used in a priming paradigm [44] and have been used to explore effects of concreteness. However, to date, the majority of the imaging studies investigating lexicality have focused on the visual modality [6, 31–33] while lexical decision tasks employing the auditory modality have been largely neglected. Critically, the visual modality has a number of limitations. Visual representation codes associated with picture-based stimuli may have a confounding effect while shallow processing has been associated with rapid orthographic analysis in written lexical decision tasks [45]. Visual lexical-semantic tasks might be able to be performed with only superficial semantic processing due to orthographic familiarity of the stimuli [46, 47] and furthermore, studies that have investigated visual lexical decision tasks have identified increased activation in the inferolateral regions which may be more attributable to grapheme - phoneme conversion routes rather than semantic processing per se [48–50].

Findings from auditory modality studies investigating lexicality have in some cases not observed activity in regions associated with phoneme to grapheme conversion [41, 51], further highlighting the benefits of using the spoken word to explore concrete and abstract processing. However, to date, the reported activation from auditory modality tasks has also not been consistent across studies [41, 51–53]. One possible reason for this variability is the type of nonword baseline used to discriminate phonological and semantic based processes. Methods used to create nonwords can vary enormously and result in the formation of very different types of nonwords [54] which may engage different processing mechanisms [52]. Raettig and Kotz [52] demonstrated that lexical transparency, the point of deviation and phonotactic legality all affect how a nonword will be processed. If a nonword is too like a real word, it may activate semantic representations whereas if it is too ‘unwordlike’ it will be dismissed prior to any phonological level processing. They conclude that opaque pseudowords are the preferred type of nonword stimuli. While opaque pseudowords are able to be processed phonologically as legal real words, lexical access is prevented and consequently any semantic based processing will be minimised [52].

As such, the aim of this study was to investigate the neural basis of the concreteness effect during spoken word recognition and employ a lexical decision task with a novel pseudoword condition. The method used in this study to generate the pseudowords is comparable to the Balota et al. study [55] in that the nonwords were created by reordering the real word stimuli. However the stimuli employed in the Balota et al. [55] study were monosyllabic words of 1 to 3 letters whereas this present study used polysyllabic words which were developed according to the criteria described in Raettig and Kotz [52] and Valdois et al. [56]. This method involved rearranging the syllables from the real word conditions while ensuring that the constituent position of each syllable remained constant [56]. As a result, the pseudowords were comparable to the real words in terms of phonology, length and legality but opaque enough to prevent elicitation of conceptual semantic representations [52]. Consequently, we predicted that the use of a robust baseline would enable observation of discrete brain activation associated with concrete and abstract spoken word processing. In addition, by making the pseudowords as close to real words as possible, discrimination between the word types was made as difficult as possible thereby increasing any semantic based activation associated with the real word conditions [34]. We examined activity in regions of interest (ROIs) selected from the meta-analysis of Binder et al. [42] to explore whether the reported ‘perceptual’ and ‘verbal’ regions could be reliably activated during spoken word recognition of concrete and abstract words using an auditory lexical decision task.

## Methods

### Materials

The stimuli consisted of 60 English real words and 60 phonologically legal pseudowords. The 60 real words comprised 30 concrete, high imageability polysyllabic nouns (e.g. wallet, hospital) and 30 abstract, low imageability polysyllabic nouns (e.g. saga, rarity). Stimuli in both real word conditions were controlled for the following variables; (i) spoken word frequency (SpFreq), (ii) written word frequency (WFreq), (iii) phoneme length, (iv) phonological neighbourhood density (PND), (v) concreteness (Concr), (vi) imageability (Img) and (vii) number of syllables (Refer Table 1 for statistical significance and stimuli characteristics). There were no significant differences (p > 0.05) between the two real word conditions on any of the variables except for (v) concreteness and (vi) imageability. Average durations for the stimuli were analysed using the Kruskal-Wallis test [57] and were not significantly different across conditions (p > 0.05) with mean durations of 724 ms (SD: 105 ms) for concrete, 772 ms (SD: 119 ms) for abstract and 780 ms (SD: 93 ms) for pseudowords.

**Table 1:** **Summary statistics for the two stimulus conditions (Mean (SD)) for a range of variables, plus the significance from a t-test comparing conditions**

Condition	SpFreq CELEX ^1^	Phonemes MRC ^2^	WFreq CELEX ^1^	PND ELP ^3^	Concr MRC ^2^	Img MRC ^2^	Syllable MRC ^2^
Concrete	30.2 (46.4)	6.1 (1.4)	45.9 (45.3)	1.0 (1.2)	586.9 (27.4)	600.5 (17.2)	2.47 (0.5)
Abstract	39 (31.3)	6.2 (1.4)	52.4 (38.7)	0.77 (0.9)	300.03 (42.2)	328.9 (31.3)	2.47 (0.5)
*p*	*.397 ns*	*.780 ns*	*.553 ns*	*.345 ns*	*<.0001*	*<.0001*	*1.000 ns*

^1^N-Watch database [58].

^2^MRC psycholinguistic database [59].

^3^English Lexicon Project [44].

Sixty pseudowords, matched for phonemes and syllables were created from the 60 real words. All the real words were segmented according to their syllable boundaries, taken from the MRC database [59]. The syllables for each word were then recombined but kept in their original constituent positions [56] in order to control for syllable frequency. Thus sixty, phonologically legal, opaque pseudowords of 2 and 3 syllables were created (e.g. culief, esiter) in accordance with Raettig and Kotz [52]. Syllable boundaries were checked to ensure legality. Any mix of syllables that resulted in an illegal combination or a word that resembled a real word was not selected for inclusion. All stimuli were digitally recorded with a Rode NTK condenser microphone in a soundproofed room using a trained, female native English speaker who was not made aware of the study purpose.

### Participants

Twenty-six healthy young participants aged between 18 and 35 years were recruited to the study. All participants had English as a primary language, were right-handed as indicated by the Edinburgh Handedness Inventory [60] and had sufficient vision and hearing to perform the task. None of the participants reported any neurological disease or disorder, mental illness, head trauma, alcoholism or cerebral tumour. Structural scans from all participants were reviewed by a neuroradiologist for abnormalities. As a result, three of the 26 participants were identified as having an incidental finding of clinical significance and were not included in the analysis. Six additional participants were excluded due to subject compliance and technical issues leaving a total of 17 subjects in the analysis (9 female, 8 male; mean age 27, SD: 5.1). The study received approval from the Queensland Health Ethics Committee, University of Queensland Medical Research Ethics Committee and site specific approval from the Royal Brisbane and Women’s Hospital Ethics Committee. Full written consent was obtained from all participants and each received a $30 reimbursement.

### Procedure

**Figure 1:**
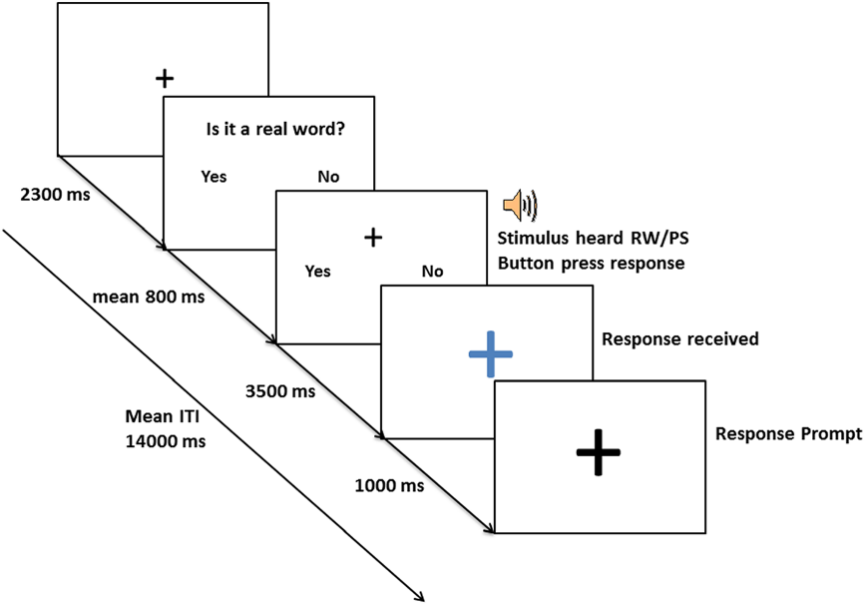
**Acquisition sequence for auditory lexical decision task (RW – real word, PS – pseudoword).**

The experiment consisted of 120 trials presented in an event-related design with two individual runs completed in the same session. Prior to the scanning session, the task was explained to each participant and examples practised until at least an 80% success rate was achieved. Once inside the scanner, the button press was placed in the participant’s left hand and reminders given about the correct orientation for response, with a left button for ‘yes’ to respond to real words and a right button for ‘no’ to respond to pseudoword stimuli. The left hand was used to enable comparisons to be made with a future-planned companion study on people with post-stroke aphasia performing the same task. At the start of each stimulus presentation, a small black fixation cross was presented visually in 48 point font for 2.3 s, along with the instruction ‘Is it a real word?’. Each real word or pseudoword was presented auditorily with the mean length of presentation lasting an average of 764 ms (SD: 105 ms). After hearing each stimulus, subjects had 3.5 s to respond. During the response/sound window a black “+” was on the screen plus Yes/No indicating the correct orientation for response. Once a response had been received, a large blue cross (84 point font) appeared on the screen. After 1 s, this changed to a black cross (84 point font) which remained visible until the start of the next trial/event as detailed in Figure 1.

A long inter-trial interval (jittered between 10-18 s, mean 14 s) was employed to allow for the delayed hemodynamic response function observed with stroke patients [61, 62] and to enable future comparison with a planned companion study on individuals with post-stroke aphasia. Order of presentation within each run was pseudorandomised with no more than four items from any one condition (concrete real words, abstract real words and pseudowords) being presented in succession and never more than four words or pseudowords in a row. Additionally, real words and pseudowords which shared constituent syllables were never presented in consecutive order. Five different pseudorandomised orders were applied to reduce order effects across participants. All stimuli were presented binaurally using MR confon headphones (MR Confon GmbH, Magdeburg, Germany) and responses recorded with an MR compatible button box (Current Designs Inc, Philadelphia PA). Prior to the scan, subjects were reminded to keep their eyes open and to look at the fixation cross in order to prevent eye movement.

### Data acquisition

Structural and functional images were collected on a Siemens Trio (3 T; Siemens Erlangen) at the Royal Brisbane and Women’s Hospital. The design of the fMRI task was a pseudo-randomised event-related design consisting of two runs of approximately 14.4 minutes administered across one session. Gradient echo EPI images with BOLD sensitivity were acquired during each of the runs, resulting in 390 brain volumes, 36 × 3 mm slices, with a 0.3 mm gap, FOV 220x220mm, flip angle 90, matrix 64x64, TE 30 ms, TR 2210 ms. A high resolution 3D structural T1 weighted MP-RAGE image was also acquired ((0.9 mm)^3^ resolution; TE/TR 2.4/1900 ms TI 900 ms).

### Image processing

Images were processed and analysed using statistical parametric mapping software (SPM8, (Wellcome Trust Centre for Neuroimaging; http://www.fil.ion.ucl.ac.uk/spm)) with MATLAB 2009a (The MathWorks Inc., Natick, MA). To allow for steady state magnetization, five dummy scans were acquired at the start of each run and excluded from analysis. INRIAlign [63] was used for spatial preprocessing of the image time series and to correct for motion artefacts. Registration of the time series mean EPI image in Run 1 and Run 2 was coregistered with the T_1_ image which had been acquired in the same scan session. The T_1_ image was segmented using New Segment [64]. A DARTEL template was created from all participants and each subject normalized to it [65]. These transformations were applied to the realigned EPI time series before an 8 mm full-width half-maximum (FWHM) Gaussian kernel was applied to spatially smooth the normalised volumes (3.0 × 3.0 × 3.0 mm^3^).

### Data analysis

A general linear model analysis using a hemodynamic response function with derivatives was used for the fixed effects analysis per subject. Conditions in Run 1 and Run 2 were combined in the design matrix. Due to the small numbers of errors (average 2 per subject), incorrect trials were excluded from the time series. Realignment parameters in 6 degrees of freedom were included as regressors of no interest. Covariates were created to model the BOLD signal change associated with the different event trials and contrasts included concrete – abstract, concrete – pseudoword and abstract - pseudoword. The mean corrected reaction time was included as a parametric modulation per condition, similar to Binder et al. [6], to include a measure of time spent on task such that comparisons of concrete to abstract to pseudowords did not include any BOLD response due to variability in reaction time.

A random effects analysis of the group was carried out on each condition. Differences in BOLD signal between the three conditions of concrete, abstract and pseudoword were evaluated using a regression analyses. The FWHM was calculated from the square root of the residuals (with 3dFWHMx; Analysis of Functional Neuroimages [66]) and used as input to 3dClustSim to calculate a cluster threshold correction for multiple comparisons. Adopting a height threshold of p < 0.001 uncorrected for the whole brain analysis, a FWE (familywise error) rate of p < 0.05 was achieved with a minimum cluster threshold of 45 contiguous voxels. The probabilistic cytoarchitectonic maps from the Anatomy Toolbox [67] were used to identify the neuroanatomical location of peak maxima for the specific contrasts.

**Figure 2:**
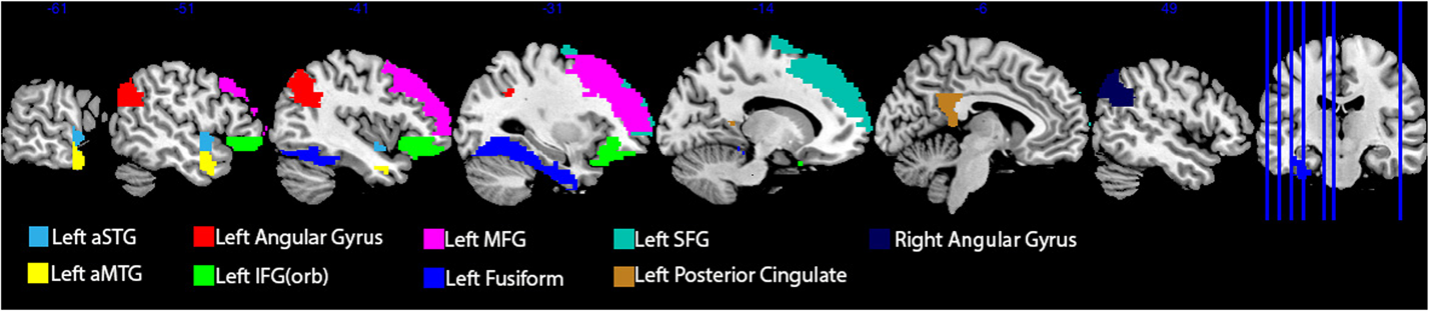
**Nine anatomical ROIs. Light blue – aSTG, yellow – aMTG, green – IFG(orb), pink – MFG, turquoise – SFG, blue – Fusiform, red – left AG, dark blue – right AG, gold –posterior cingulate.**

Region of interest (ROI) analyses were carried out based on a priori hypotheses about hemispheric lateralization differences associated with the processing of concrete and abstract words and findings from the Binder et al. meta-analysis [42]. As a result, a total of nine anatomical ROIs were selected (see Figure 2). Six of these ROIs were associated with concrete processing and were created in the left and right AG, left superior frontal gyrus (SFG), left middle frontal gyrus (MFG), left posterior cingulate and left fusiform. Binder et al. [42] also reported increased activation for abstract concepts in the left IFG (mainly pars orbitalis) and left aSTS. As such, three further ROIs were created to explore potential activity associated with abstract word processing. ROIs were created in the anterior superior temporal gyrus (aSTG) and anterior middle temporal gyrus (aMTG) to enable investigation of the aSTS and a third ROI was created in the left pars orbitalis.

ROIs were created by using the IBASPM 116 Human Atlas in WFU PickAtlas [68, 69] in SPM8 (Wellcome Trust Centre for Neuroimaging; http://www.fil.ion.ucl.ac.uk/spm). Anterior ROIs (aSTG and aMTG) were subdivided based on the classification of Indefrey and Levelt [70] of y < -7. MarsBaR [71], a region of interest toolbox for SPM8, was used to extract the percentage mean signal change for each participant for each ROI. Group statistical analyses were then performed using SPSS Statistics version 21 (IMB; Armonk, New York, USA). A repeated measures analyses of variance (ANOVA) with factors of condition (3) x ROI (9) was performed. Huynh-Feldt corrections [72] were applied when the assumption of sphericity was not met and are reported throughout with the original degrees of freedom. Testing for pairwise differences between conditions (concrete – abstract, concrete – pseudoword, abstract – pseudoword) in all nine ROIs was performed with an adjusted p level using Benjamini and Hochberg’s correction for multiple comparisons [73].

## Results

### Behavioural results

Reaction time data was calculated from stimulus onset. Only correct responses were analysed, with trials less than 100 ms removed. There was a significant main effect for condition (*F*(2, 1999) = 91.582, *p <* 0.0001). Participants responded fastest to concrete words followed by abstract words then pseudowords (see Table 2) with a significant difference between each condition (all p < 0.001). There was no statistically significant difference in accuracy between conditions (p = 0.386).

**Table 2:** **Behavioural results**

Condition	Reaction time (SE) (ms)	% Accuracy
Concrete	1187.19 (15.89)	98.6 (0.006)
Abstract	1262.99 (16.00)	97.5 (0.006)
Pseudoword	1433.81 (11.26)	98.1 (0.004)

### Anatomical region of interest analyses

**Figure 3:**
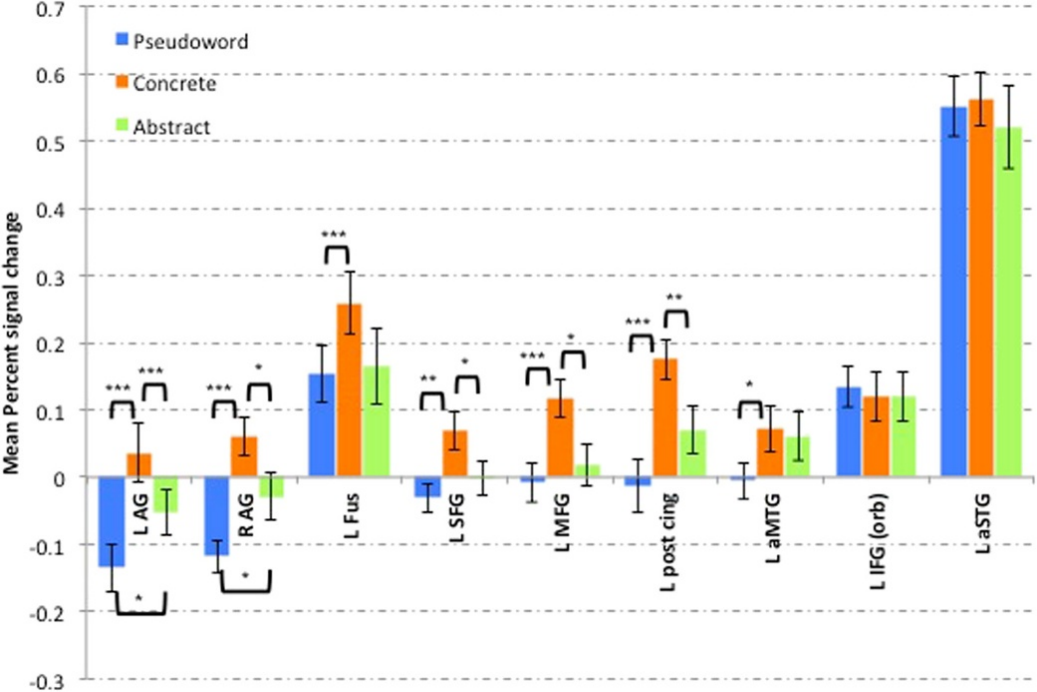
**Percent signal change from the regions of interest.** * p < 0.05; ** p < 0.005; *** p < 0.0001. (Abbreviations: L AG = left angular gyrus; R AG = right angular gyrus; L Fus = left fusiform, L SFG = left superior frontal gyrus; L MFG = left middle frontal gyrus; L post cing = left posterior cingulate; L aMTG = left anterior middle temporal gyrus; L IFG (orb) = left inferior frontal gyrus (pars orbitalis); L aSTG = left anterior superior temporal gyrus).

Results from the ROI analyses are shown in Figure 3 with the mean percent signal change of BOLD signal for the three conditions in each of the ROIs. There was a main effect of condition for the left AG, *F*(1.225, 19.601) = 18.248*, p <* 0.0001. Increased BOLD signal was observed for concrete (p < 0.0001) and abstract (p = 0.008) compared to pseudowords and concrete compared to abstract words (p < 0.0001). There was a main effect of condition for the right AG, *F*(2, 15) = 21.15, *p <* 0.0001 and increased BOLD signal for concrete (p < 0.0001) and abstract (p = 0.025) compared to pseudowords. Concrete words also elicited increased BOLD signal compared to abstract words (p = 0.007).

A main effect of condition was observed for left SFG *F*(2, 15) = 8.812, *p =* 0.003. There was increased BOLD signal for concrete compared to abstract (p = 0.049) and pseudoword (p = 0.004). There was a main effect of condition for left MFG *F*(2, 15) = 9.897, *p =* 0.002 and increased BOLD signal for concrete compared to abstract (p = 0.008) and pseudoword (p < 0.0001). There was also a main effect of condition for left posterior cingulate *F*(2, 15) = 12.579, *p =* 0.001 with increased BOLD signal for concrete compared to abstract (p = 0.004) and pseudoword (p < 0.0001). A main effect of condition in the left fusiform *F*(1.346, 21.529) = 4.811, *p =* 0.03 was also seen with increased BOLD signal for concrete compared to pseudowords (p < 0.0001).

The left IFG (pars orbitalis), aSTG and aMTG ROIs were included to investigate association with abstract word processing [42]. Of these only left aMTG showed a main effect for condition (*F*(2, 15) = 7.49*, p =* 0.006), but with increased BOLD signal for concrete compared to pseudowords (p = 0.025).

### Whole brain analyses

**Figure 4:**
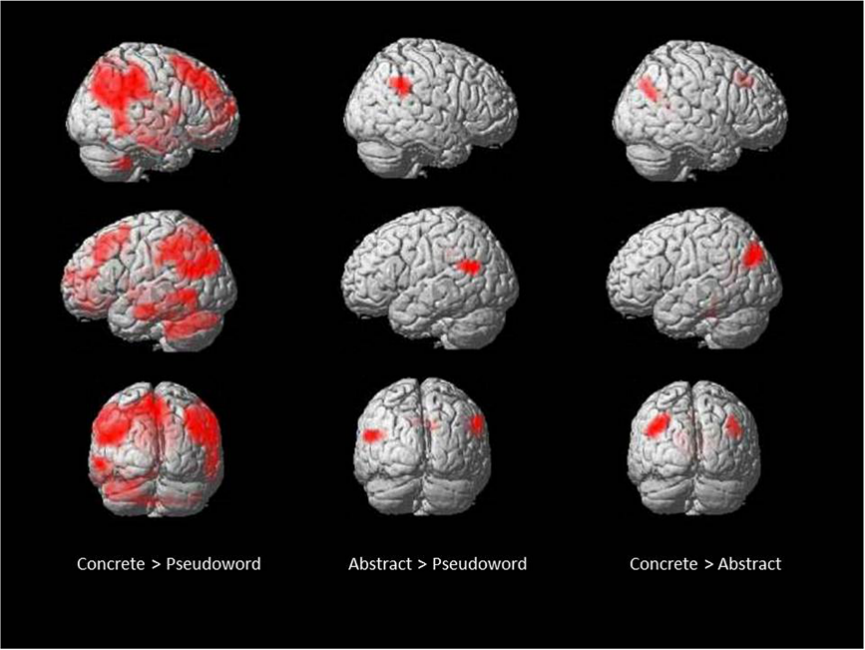
**Whole brain results.** Significant peak activations for group whole brain analyses in each condition are shown in red on a rendered brain. Surface structures are in darker shades of red. Significance was determined by a p < 0.001 probability threshold and 45 voxel cluster threshold.

The whole brain results for concrete greater than pseudoword, abstract greater than pseudoword and concrete greater than abstract are shown in Table 3 and Figure 4. No cortical regions were significantly more activated for abstract greater than concrete words. There was a bilateral pattern of activity for both concrete and abstract words when compared to pseudowords. The right precuneus was the sole region that was commonly activated by both concrete greater than pseudoword and abstract greater than pseudoword contrasts and this was more extensive for concrete than abstract words, extending into right AG and occipital regions. In the direct contrast between concrete and abstract, a bilateral pattern of activity was elicited for concrete words. However, this bilateral activity appeared to be slightly more left-lateralised.

**Table 3:** **Whole brain regions showing significant BOLD peak activation**

Contrast	Structure	x	y	z	Volume	Z-score
Concrete > Pseudoword	Right precuneus	7	-50	47	6101	6.14
	Right hippocampus	32	-32	-7	609	4.93
	Left MFG	-32	25	54	341	4.49
	Left ITG	-54	-14	-25	105	4.26
Abstract > Pseudoword	Left MTG	-54	-58	18	82	4.37
	Right SMG	50	-40	32	62	4.04
	Right precuneus	14	-50	32	58	3.86
Concrete > Abstract	Right mid occipital g	40	-65	29	85	4.89
	Left IPC (PGP)^1^	-47	-76	29	141	4.53
	Left calcarine g	-7	-58	7	349	4.47
	Left fusiform	-22	-36	-18	51	4.47
	Right MFG	25	22	40	53	4.16

Peak activations for whole brain analyses for all participants in each condition (p < 0.001 probability threshold and 45 voxel cluster threshold). MFG = middle frontal gyrus, ITG = inferior temporal gyrus, MTG = middle temporal gyrus, SMG = supramarginal gyrus, g = gyrus.

^1^See Caspers et al. [74] for details of this area.

### Summary of results

There was a concreteness effect in terms of faster speed of response for concrete words compared to abstract words. Anatomical ROI analyses revealed significant increased signal for concrete words compared to both abstract and pseudowords in left and right AG, left SFG*,* left MFG, left posterior cingulate. Significant signal differences were also observed in the left fusiform and left aMTG for concrete words but only for the concrete – pseudoword contrast. The AG was the sole brain region which elicited significant differences in activity between all three conditions, with concrete words eliciting greater activation than both abstract and pseudowords. Abstract words also produced greater activity than pseudowords in bilateral AG. Greater activity for abstract words compared to concrete words was not observed in any of the nine ROIs. Whole brain results showed bilateral activity for concrete and abstract words when compared to pseudowords. Bilateral activation was also seen for concrete words when directly compared to abstract words.

## Discussion

The aim of this study was to investigate brain activity associated with recognising spoken concrete and abstract words. We predicted that concreteness effects would be reflected in reaction time data and accuracy of response. We also predicted that the use of an opaque pseudoword condition would provide a robust baseline against which to measure differential brain-related activity elicited directly in response to concrete and abstract spoken word recognition. We anticipated that concrete words would elicit increased brain activity in bilateral brain regions while abstract words would elicit greater activity in left-lateralised language processing networks. The present study revealed bilateral activity for concrete words, however we did not see a left-lateralised pattern of activity for abstract words. Our results confirm the involvement of the angular gyrus, posterior cingulate and dorsolateral prefrontal cortex in retrieving concrete versus abstract concepts during spoken word recognition, consistent with previous meta-analyses on concrete and abstract processing based primarily on visually presented words [42, 43]. Overall, our findings suggest that heteromodal association areas were activated when recognising spoken concrete and abstract words consistent with the view that these regions interface with a critical set of modality-specific representations.

The behavioural results showed that concrete items were responded to significantly faster than both abstract and pseudoword conditions and the abstract condition was significantly faster than the pseudoword condition. The observed increased efficiency for concrete over abstract words is in accordance with the two dominant theoretical accounts used to explain effects of concreteness; dual coding theory [5] and context availability theory [17, 75] which both predict a processing advantage for concrete words. There was no difference in accuracy across the three conditions which was most likely due to a ceiling effect.

Of the nine ROIs selected to investigate concrete and abstract word processing, the right and left AG proved to be the sole brain region that showed significant differences in activation between all three contrasts with concrete words eliciting greater activity than both abstract words and pseudowords and abstract words eliciting stronger activation than pseudowords. Increased activation for concrete items in the right AG has previously been reported previously in visual word processing studies investigating concrete and abstract words [6, 39]. These findings provide support for dual coding theory which predicts that concrete words will recruit additional image-based codes represented in the right hemisphere. However, our results also showed stronger activation in the left AG for concrete items compared to abstract words which is inconsistent with dual coding theory. Rather, it is more consistent with a single system model of processing such as the context availability theory. This model suggests that concrete words access the same verbal regions as abstract words but to a greater degree due to the larger amount of associated context available to concrete words. As such while the context availability theory does not preclude right hemisphere involvement, the verbal language-based left hemisphere regions are more likely to be recruited. The left AG has been associated with general semantic retrieval processes [76] and was the region which was most reliably activated across the 120 studies in the Binder et al. semantic meta-analysis [42] as well as having the largest number of activation foci for concrete words specifically [42].

The ROI analysis also revealed greater activity for concrete versus abstract words in the left posterior cingulate, SFG and MFG. The posterior cingulate has previously been described as a connector hub, structurally linking other cortical and subcortical brain networks [77]. Binder et al. [42] suggest that this region is implicated in many semantic based tasks as a result of the nature of episodic encoding. Words that have a richer set of concepts and associations such as concrete items will evoke enhanced episodic encoding [42] while activation of abstract words will be less strong as they have weaker associations with specific episodic memories.

Significantly greater activity for concrete compared to abstract words in the left MFG and SFG also suggests a critical involvement of these frontal regions in the processing of concrete information. However, the specific role of these regions in semantic conceptual processing is not well understood and their activation in language-based tasks is commonly thought to be in response to more executive type task demands. The MFG and SFG have previously been associated with working memory [78] and have been implicated in a distributed network monitoring and facilitating comprehension [79, 80]. The MFG has been associated specifically with auditory lexical decision tasks [81] and controlled cognitive processing [82]. Whole brain results also indicated an involvement of the right MFG for the concrete greater than abstract contrast and this region has previously been associated with auditory attention and comprehension [81, 83] and monitoring [84].

Previously, executive task-related engagement of frontal regions has been associated with increased effort and a corresponding increased response time, however the findings in this present study do not support this view as our behavioural results demonstrate clear involvement of these regions in concrete word processing without any associated increase in time on task. It is possible these frontal regions are recruited to monitor activation of the word candidate in the associated lexical-semantic network while a decision on lexicality is made [81], however this does not explain why significant activity was observed for concrete words only. An alternative proposal is that these frontal regions are implicated in the creation, integration and manipulation of a strong mental representation as sensory-motor information associated with concrete concepts is activated within the language processing network [3, 85]. This interpretation should be treated with caution however, given that our whole brain analyses did not identify increased activity in other regions associated with sensory-motor processing for this contrast.

ROI analyses of both the left fusiform gyrus and aMTG elicited additional activation in the concrete greater than pseudoword contrast. The fusiform has been referred to as a basal temporal language area [86] and our findings for concrete words are consistent with a role that this region plays in visual imagery associated with language processing. The fusiform has previously been associated with both lexical decisions [32] and auditory semantic retrieval processes [87]. Discrete activation in this brain region relating to concrete word processing has also been observed in studies investigating imageability associated with concrete and abstract word processing [27, 36–38] and this has been attributed to the greater levels of visual imagery associated with concrete concepts [27, 31, 36, 38]. Wise et al. [36] suggest that once a word has been perceived as real, the fusiform serves to encode semantic representations (in terms of episodic and semantic memory) associated with the verbal input and this region may therefore act as a lexical-semantic store [88].

The aMTG was examined as an ROI to explore the aSTS, which was reliably activated by abstract words in the Binder et al. meta-analysis [42]. In order to ensure complete coverage of the aSTS, both the aMTG and aSTG were included as ROIs although neither region elicited any increase in activity for abstract words. On the contrary, our results for aMTG showed a significant increase in activity for concrete words when compared to pseudowords. The anterior MTG has previously been associated with the access of conceptual representations as they become increasingly more semantically specific [89] and the anterior temporal lobe has been described as an amodal semantic hub, implicated in the integration of conceptual and semantic information [90, 91]. Lesions in aMTG have been associated with lexical-semantic deficits and category specific impairments [92, 93]. The literature is mixed on the results of aMTG lesions and concreteness effects with Jefferies et al. [12] showing a processing advantage for concrete over abstract words while other studies have demonstrated a reverse concreteness effect (i.e. greater preservation of abstract words) [94, 95].

Our results clearly demonstrate that lateral and ventral temporal regions were engaged in lexical-semantic processes but only for the concrete greater than pseudoword contrast. No differences were observed in these regions for either the concrete greater than abstract or abstract greater than pseudoword contrasts. As such, we tentatively propose that these regions may serve as lexical-semantic stores prior to subsequent processing and that the selective activation observed for concrete words when compared to pseudowords may be due to equivalent activation in these regions from processing nonverbal codes associated with abstract words and processing verbal codes associated with opaque pseudowords. To expand on the first point, we suggest that abstract words like concrete words are in fact associated with a set of nonverbal imaginal representations although they are just more weakly represented than the nonverbal representations associated with concrete words. For instance, a word such as *charity* might be associated with nonverbal, imaginal associative knowledge such as a collection tin or a red cross sign [36] and will therefore activate a set of nonverbal associations, albeit more weakly than concrete words which have strong imaginal referents attached to them. As a result, potential differences in brain-related activity in these regions related to concrete and abstract conceptual processing will be reduced.

Secondly, no significant differences in activation between abstract and pseudowords was observed in these lateral and ventral regions. Indeed, the lack of activation differences associated with abstract and pseudoword processing was a common finding in this study. Of the nine ROIs, only bilateral AG elicited a difference between abstract and pseudoword processing. We suggest that this finding may be due to the type of pseudowords employed in this study and the verbal codes attached to them. The opaque pseudowords shared features common to both the abstract and concrete words such as phonemes and syllables which enabled them to be processed in the same lexical networks as the real words [96]. However, the opaqueness minimised any word specific knowledge and subsequent semantic based activity [52]. As such, the opaque pseudowords activated associated verbal codes as the lexicon was searched for real word matches, resulting in brain activity similar to that evoked during the auditory presentation of abstract words to a degree and reducing any activation differences associated with the spoken word recognition of abstract and pseudowords. However, unlike the abstract words, nonverbal representations were not associated with the pseudowords as the stimuli were meaningless and imaginal codes therefore non-existent. Thus, we suggest that where there were differences between concrete and pseudoword contrasts only, such as in the lateral and temporal regions, these are likely due to the strong nonverbal (conceptual) representations or features associated with concrete words, which are only weakly represented in abstract words and not associated at all with the opaque pseudowords.

In summary, our findings demonstrate that brain activity was reliably elicited during this auditory lexical decision paradigm in most of the regions previously showing activity for concrete word processing using visual word processing paradigms; bilateral AG, left posterior cingulate, SFG, MFG, fusiform and aMTG. In terms of the prominent theories regarding concrete and abstract word processing our findings for the recognition of spoken concrete words provide some support for both a dual representation and single system model. The selective activation of concrete words in three of the six ‘perceptual’ regions; left SFG, MFG and posterior cingulate, identified in the Binder et al. meta-analysis [42], provides support for dual coding theory which predicts that concrete words are represented in distinct neural systems which are differentially activated in response to more image-based, nonverbal codes. The additional activation for concrete items in the right AG is also consistent with dual coding theory which predicts greater recruitment of nonverbal, right hemisphere for concrete words.

However, we also observed quantitative activation differences for all three conditions in left and right AG suggesting that this brain region serves as a common zone which is differentially activated in response to both abstract and concrete terms compared to pseudowords. These findings provide support for the context availability theory which proposes that in a single system model, common regions will be implicated in real word processing but to differing degrees. Since concrete words have a richer set of contextual representations available to them than abstract words, they will elicit stronger activity than abstract words although activity will occur in common regions.

Importantly, our findings provide support for the embodied abstraction theory proposed by Binder and Desai [21] which suggests that large parts of the temporal and inferior parietal cortex serve as multimodal convergence zones and are integral in the binding of modality-specific information. Specifically, our results support the view that bilateral AG serves as a convergence zone binding supramodal input from modality-specific and heteromodal systems. Conceptual representations of concrete and abstract words will be differentially processed according to the amount of associated sensory, motor and affective representations with levels of activity in the AG responding to the amount of semantic information associated with the target word. Both concrete and abstract words will elicit greater levels of activity compared to pseudowords due to the greater amount of semantic representations attached to real words. However, concrete words will elicit stronger activity than abstract words as they are associated with stronger conceptual representations.

Lastly, the ROI and whole brain results for abstract word processing in this present study are in agreement with both dual coding and context availability theory in that neither theory proposes that greater activity should be elicited by abstract compared to concrete words. However, our results also show minimal processing differences when abstract words were compared with pseudowords and this lack of activation difference was a pattern which was consistent in all ROIs except bilateral AG. This is an interesting finding and whilst we have provided a tentative explanation for the increased activity observed for concrete but not abstract words when compared to pseudowords, the present study cannot definitively say whether the this pattern of activity can be attributed to the type of stimuli, the task employed or modality of presentation. Future studies investigating spoken word recognition of concrete and abstract words will need to consider possible effects of the verbal and nonverbal, imaginal codes likely associated with abstract conceptual representations.

Whilst this study has provided a number of findings regarding the processing of spoken concrete and abstract words during a lexical decision task, we acknowledge that there are some limitations with the stimuli selection employed. Firstly, we were unable to control for the first phoneme across the conditions and as such, this needs to be considered as a possible confound and the results interpreted accordingly. Secondly, the abstract stimuli selected for use in this study were also of very low imageability and it may be helpful in future studies to attempt to match for imageability across both concrete and abstract conditions although this is challenging.

## Conclusion

Findings from the ROI analyses in this study demonstrate that the recognition of spoken concrete words reliably activates a wide network of brain regions in the AG bilaterally, left posterior cingulate and left dorsolateral prefrontal cortex more than both abstract and pseudowords. Concrete words also activated regions in the left fusiform and left anterior MTG but this activation was only elicited for the concrete greater than pseudoword contrast. Our findings further define possible convergence zones proposed to bind supramodal input from modality-specific and heteromodal systems, and confirm the role of these regions in processes beyond initial modality specific lexical processing.

## Abbreviations

AG: Angular gyrus
IFG: Inferior frontal gyrus
aSTS: Anterior superior temporal sulcus
MTG: Middle temporal gyrus
STG: Superior temporal gyrus
ROIs: Regions of interest
fMRI: Functional magnetic resonance imaging
EPI: Echo planar imaging
BOLD: Blood-oxygen level dependency
ROI: Region of interest
SFG: Superior frontal gyrus
MFG: Middle frontal gyrus
aSTG: Anterior superior temporal gyrus
aMTG: Anterior middle temporal gyrus.
